# Identification of an Autophagy-Related Pair Signature for Predicting Prognoses and Immune Activity in Pancreatic Adenocarcinoma

**DOI:** 10.3389/fimmu.2021.743938

**Published:** 2021-12-09

**Authors:** Qian Zhang, Liping Lv, Ping Ma, Yangyang Zhang, Jiang Deng, Yanyu Zhang

**Affiliations:** ^1^ Institute of Health Service and Transfusion Medicine, Beijing, China; ^2^ Beijing Key Laboratory of Blood Safety and Supply Technologies, Beijing, China

**Keywords:** pancreatic adenocarcinoma, prognostic model, small molecule compounds, autophagy-related gene pairs, tumor immune activity

## Abstract

**Background:**

Pancreatic adenocarcinoma (PAAD) spreads quickly and has a poor prognosis. Autophagy research on PAAD could reveal new biomarkers and targets for diagnosis and treatment.

**Methods:**

Autophagy-related genes were translated into autophagy-related gene pairs, and univariate Cox regression was performed to obtain overall survival (OS)-related IRGPs (P<0.001). LASSO Cox regression analyses were performed to construct an autophagy-related gene pair (ARGP) model for predicting OS. The Cancer Genome Atlas (TCGA)-PAAD cohort was set as the training group for model construction. The model predictive value was validated in multiple external datasets. Receiver operating characteristic (ROC) curves were used to evaluate model performance. Tumor microenvironments and immune infiltration were compared between low- and high-risk groups with ESTIMATE and CIBERSORT. Differentially expressed genes (DEGs) between the groups were further analyzed by Gene Ontology biological process (GO-BP) and Kyoto Encyclopedia of Genes and Genomes (KEGG) analyses and used to identify potential small-molecule compounds in L1000FWD.

**Results:**

Risk scores were calculated as follows: ATG4B|CHMP4C×(-0.31) + CHMP2B|MAP1LC3B×(0.30) + CHMP6|RIPK2 ×(-0.33) + LRSAM1|TRIM5×(-0.26) + MAP1LC3A|PAFAH1B2×(-0.15) + MAP1LC3A|TRIM21×(-0.08) + MET|MFN2×(0.38) + MET|MTDH×(0.47) + RASIP1|TRIM5×(-0.23) + RB1CC1|TPCN1×(0.22). OS was significantly shorter in the high-risk group than the low-risk group in each PAAD cohort. The ESTIMATE analysis showed no difference in stromal scores but a significant difference in immune scores (p=0.0045) and ESTIMATE scores (p=0.014) between the groups. CIBERSORT analysis showed higher naive B cell, Treg cell, CD8 T cell, and plasma cell levels in the low-risk group and higher M1 and M2 macrophage levels in the high-risk group. In addition, the results showed that naive B cells (r=-0.32, p<0.001), Treg cells (r=-0.31, p<0.001), CD8 T cells (r=-0.24, p=0.0092), and plasma cells (r=-0.2, p<0.026) were statistically correlated with the ARGP risk score. The top 3 enriched GO-BPs were signal release, regulation of transsynaptic signaling, and modulation of chemical synaptic transmission, and the top 3 enriched KEGG pathways were the insulin secretion, dopaminergic synapse, and NF-kappa B signaling pathways. Several potential small-molecule compounds targeting ARGs were also identified.

**Conclusion:**

Our results demonstrate that the ARGP-based model may be a promising prognostic indicator for identifying drug targets in patients with PAAD.

## Introduction

Pancreatic adenocarcinoma (PAAD) is a cancerous tumor that spreads quickly and has a poor prognosis. It has a low incidence rate, but because of its high mortality rate, its incidence and death rates are nearly identical (1). Pancreatic cancer is the fourth leading cause of cancer-related death in developed countries according to epidemiological data, and some data suggest that it may overtake lung cancer as the second most common cause of cancer-related death by 2020 (2, 3). Because patients with pancreatic cancer have poor long-term prognoses, it is vital to construct a prognosis prediction model and establish tailored diagnosis and treatment plans based on the model.

Autophagy is a highly conserved intracellular degradation process by which cells remove nonessential and dysfunctional components. It is a dynamic process that includes the autophagosome induction, nucleation, double membrane growth and closure, and finally, fusion with the lysosome, and it serves as an alternative energy source during periods of metabolic stress to maintain homeostasis and viability (4). Abnormal autophagy has been reported to be associated with the pathogenesis of a variety of diseases, including malignant tumors (5, 6). Autophagy has been mainly described as a mechanism of resistance to various cancer treatments, such as chemotherapy (7), targeted therapy (8) and immunotherapy (9). However, it seems to exhibit bidirectional regulation of tumorigenesis. For example, a low level of autophagy promotes the initiation of early-stage cancer in gynecological cancers, while a high level of autophagy promotes tumor cell survival in a nutrient-deficient microenvironment (10, 11).

In pancreatic cancer, a high basal rate of autophagy has been described in several human pancreatic ductal adenocarcinoma (PDAC) cell lines, and autophagy was shown to be upregulated in the later stages of progression of premalignant lesions in PDAC but not in normal pancreatic ducts (12–14). Notably, autophagy has been shown to be required in the tumor response to various anticancer therapies (15, 16), including chemotherapy and radiotherapy, which can induce autophagy on their own and potentiate the effect of immunotherapy (17, 18). Another study of PAAD revealed that the role of enhanced autophagy/lysosome function in immune evasion was exerted by selective targeting of MHC-I molecules for degradation (19). However, researches regarding the contribution of autophagy in immune cells in PAAD are still limited. It is necessary to further explore the roles of autophagy and immune cell infiltration in pancreatic cancer.

In recent years, several gene expression signatures based on autophagy-related genes (ARGs) have been reported to predict the prognoses of various cancers, including non-small-cell lung cancer (20), ovarian cancer (21), breast cancer (22), and colon cancer (23). One recent study reported the prognostic role of ARGs in pancreatic cancer; however, there was a lack of validation in external databases (24). In this study, we established an autophagy-related gene pair (ARGP)-based model for predicting pancreatic adenocarcinoma prognosis. Subsequently, we reported a prognostic model based on ARGPs and verified its performance in multiple external datasets (GSE57495, GSE78229, and GSE85916). We found that the expression of ARGPs is associated with the tumor microenvironment and immune infiltration and suggested a list of several small-molecule compounds targeting ARGPs. Taken together, our results demonstrate that the ARGP-based model may serve as a promising prognostic indicator and may provide drug targets in patients with PAAD.

## Materials And Methods

### Study Design and Data Collection

**Figure 1:**
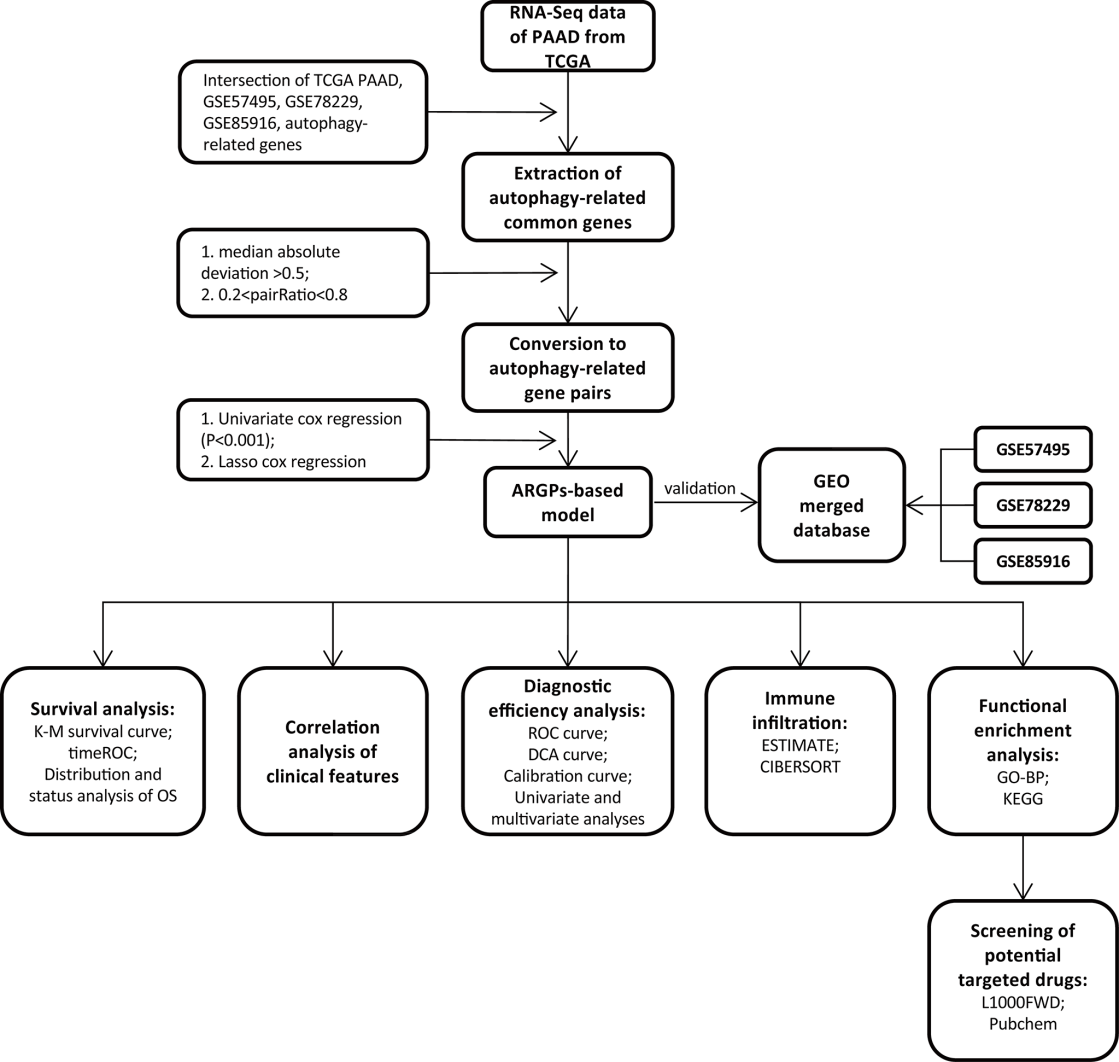
Flow chart of the overall study design.

The flow of the experimental design and data analysis is shown in **Figure 1**. Transcription profiles (FPKM) and clinical data of TCGA-PAAD patients were obtained from the UCSC Xena website (https://xenabrowser.net/datapages/) and set as the training group. Microarray datasets (GSE57495, GSE78229, and GSE85916) were downloaded from the Gene Expression Omnibus (GEO) portal (https://www.ncbi.nlm.nih.gov/geo/) and were merged as the external test group for further validation of the signature. The basic information of the selected patients from the four databases is shown in **Table 1**.

**Table 1:** Clinical data of PAAD cohort from TCGA, GSE57495, GSE78229, GSE85916.

	TCGA-PAAD	GSE57495	GSE78229	GSE85916
**Age (year)**				
<65	81	–	–	–
≥65	95	–	–	–
unknown	0	–	–	–
**Sex**				
Female	80	–	–	–
Male	96	–	–	–
**Survival status**				
Alive	88	21	14	22
Dead	88	42	35	57
**Grade**				
Grade 1	30	–	2	–
Grade 2	94	–	24	–
Grade 3	48	–	22	–
Grade 4	2	–	1	–
unknown	2	–	0	–
**Stage**				
Stage I	21	13	4	–
Stage II	145	50	45	–
Stage III	3	0	0	–
Stage IV	4	0	0	–
unknown	3	0	0	–
**T staging**				
T1	7	–	–	–
T2	24	–	–	–
T3	140	–	–	–
T4	3	–	–	–
unknown	2	–	–	–
**M staging**				
M0	79	–	–	–
M1	4	–	–	–
unknown	93	–	–	–
**N staging**				
N0	49	–	–	–
N1	122	–	–	–
unknown	5	–	–	–

ARGs were obtained from the Molecular Signatures Database (MSD, http://www.gsea-msigdb.org/gsea/msigdb/index.jsp), including the GOBP_POSITIVE_REGULATION_OF_AUTOPHAGY, GOBP_NEGATIVE_REGULATION_OF_AUTOPHAGY, KEGG_REGULATION_OF_AUTOPHAGY, REACTOME_AUTOPHAGY, REACTOME_CHAPERONE_MEDIATED_AUTOPHAGY, REACTOME_SELECTIVE_AUTOPHAGY, and WP_AUTOPHAGY gene sets (details are listed in **Supplementary Table 1**).

### Determination of Candidate ARGs and Translation of ARGPs

Candidate ARGs were further determined by intersection in multiple gene lists, including ARGs from the MSD, TCGA-PAAD, GSE57495, GSE78229 and GSE85916. The candidate ARGs were then extracted from the PAAD cohort for further analysis. ARGs with a median absolute deviation (MAD) < 0.5 were excluded to ensure prediction efficiency.

A pairwise comparison translation was performed between candidate ARG expression values to obtain an index for each ARGP in each sample. The ARGP was assigned a value of 1 if the expression of the former ARG was higher than that of the latter ARG; otherwise, the index was defined as 0. ARGPs with gene ratios (1/0 or 0/1) > 0.2 and < 0.8 were retained.

### Construction of the Prognostic Risk Model

Univariate Cox regression was performed to obtain overall survival (OS)-related IRGPs (P<0.001). Next, the LASSO Cox regression method, which is a common dimensionality-reduction method for the regression of high-dimensional data, was used to screen OS-related ARGPs without multicollinearity. An ARGP-based risk model was thereby established by multivariate Cox regression, and an equation was produced to calculate risk scores of PAAD patients with relevant ARGP indexes and respective coefficients. Finally, a formula for the risk score was established, and we calculated the risk score of each case as follows:


$$\text{RiskScore}=\sum_{t=1}^{\text{n}}\text{Coefi}\times \text{Xi}$$

Coefi indicates the correlation coefficient of each ARGP, and X indicates the level of gene expression. By performing 1-year time-dependent ROC curve analysis, the optimal cutoff value of 0.321 was set as the cutoff value, and the patients in each dataset were divided into high-risk and low-risk groups according to the cutoff.

### Evaluation of the Prognostic Capacity of the ARG Model

Evaluation was performed with R software for both the TCGA cohort and the external datasets (GSE57495, GSE78229, and GSE85916). Analysis of pancreatic cancer patient survival based on the ARGs was performed *via* the Kaplan-Meier method. Independent risk factors were identified by univariate and multivariate Cox regression analysis. Operating characteristic (ROC) curves were depicted by the timeROC package. The area under the ROC curve (AUC) was used to measure the prognostic efficiency of the model. Decision curve analysis (DCA) and calibration curves were produced with the ggDCA and rms packages, respectively.

### Protein-Protein Interaction (PPI) Network Analysis

For the included ARGs, the STRING database (https://www.string-db.org/) was used to construct the PPI network, with the parameter of confidence > 0.4. Visualization of the PPI network was performed by Cytoscape (v3.7.2) (25).

### Determination of Immune Scores, Stromal Scores, and ESTIMATE Scores

The Estimation of Stromal and Immune cells in Malignant Tumors Using Expression data (ESTIMATE) algorithm was utilized in the “estimate” R package to evaluate the ratio of the immune-stromal component in the tumor microenvironment (TME), producing three scores: the immune score (representing the level of immune cell infiltration), stromal score (representing the number of stroma), and ESTIMATE score (representing the sum of both). A higher score indicated a larger ratio of the corresponding component in the TME. Expression of potential immune checkpoint genes was also determined according to previous literature (26–35).

### Estimation of Immune Cell Infiltration

To evaluate immune cell infiltration, CIBERSORT was used to quantify 22 tumor-infiltrating immune cell subgroups between the two groups. The relationships between the expression of potential tumor-infiltrating immune cell subgroups and ARGPs were further examined by Spearman correlation analysis.

### Functional and Pathway Enrichment Analysis

DEGs between the groups were analyzed by the “limma” and “clusterProfiler” packages to perform Gene Ontology biological process (GO-BP) analysis and Kyoto Encyclopedia of Genes and Genomes (KEGG) pathway enrichment analysis.

### Identification of Potential Compounds

DEGs based on the ARG signature were divided into up- and downregulated gene groups and uploaded to the L1000FWD website (https://maayanlab.cloud/L1000FWD). Then, a table containing potential compounds was obtained. The results were further visualized using the PubChem website (pubchem.ncbi.nlm.nih.gov).

### Statistical Analysis

Data were analyzed by R software version 4.0.4. Data following a normal or nonnormal distribution were compared using unpaired Student’s t-test or the Wilcoxon test, respectively, and the statistical significance threshold was set at p <0.05.

## Results

### Establishment of the Prognostic Model With 10 ARGPs

**Figure 2:**
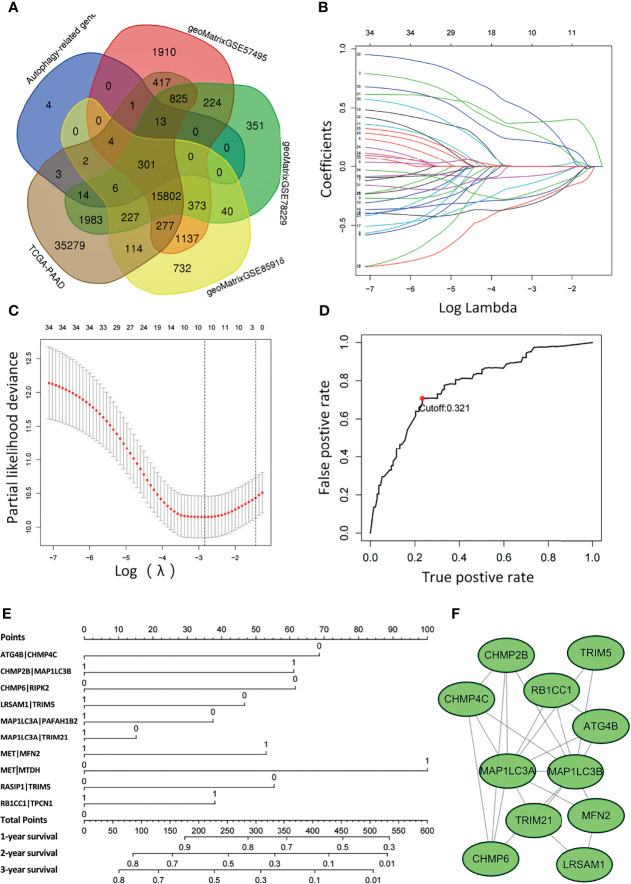
Least absolute shrinkage and selection operator (LASSO) Cox regression analysis was conducted based on the autophagy-related signature. **(A)** Venn diagram showing the 301 ARGs screened in all five cohorts. **(B)** Candidate ARGPs from the univariate Cox regression analysis were filtered by the LASSO algorithm. **(C)** LASSO coefficient profiles. **(D)** Time-dependent ROC curve of ARGPs in the TCGA-PAAD cohort (1-year AUC = 0.790, cutoff value=0.321). **(E)** Nomogram drawn to predict the 1-, 2-, and 3-year survival rates of pancreatic cancer patients. For a patient with ATG4B|CHMP4C=0, CHMP2B|MAP1LC3B=0, CHMP6|RIPK2 = 0, LRSAM1|TRIM5 = 0, MAP1LC3A|PAFAH1B2 = 0, MAP1LC3A|TRIM21 = 1, MET|MFN2 = 1, MET|MTDH=1, RASIP1|TRIM5 = 1, RB1CC1|TPCN1 = 1, the final total scores are 68+0+61+46+37+0+53+100+0+37 = 402, corresponding to a 1-year survival rate of 60%, a 2-year survival rate of 15%, and a 3-year survival rate of 10%. **(F)** The ARGs constituting the model were mapped using Cytoscape software.

**Figure 1** displays the flow chart of our study process. First, the gene expression matrix was extracted from four different PAAD cohorts, and then the intersecting genes among all datasets were identified. Candidate ARGs were selected by overlapping the intersecting genes with 348 ARGs from the Molecular Signatures Database, and a total of 301 ARGs were selected as candidate genes for further analysis (**Figure 2A** and **Supplementary Table 2**). After establishing the values of ARGPs, which were calculated according to the screened ARGs, we obtained a final set of 4441 ARGPs.

To determine the relationship between OS outcomes and ARGPs, univariate Cox regression analysis was performed and recognized 35 ARGPs that were noticeably correlated with patient prognoses. To identify robust ARGPs, we used LASSO Cox regression analysis in the TCGA-PAAD cohort. After the model reached the minimum lambda, prognostic autophagy-related gene pairs with 10 components were built (**Figures 2B, C**). We selected an optimal cutoff value of 0.321 for the IRGP index to predict PAAD prognoses using 1-year time-dependent ROC curve analysis (**Figure 2D**). With the determined cutoff value as the threshold, patients in the TCGA dataset were divided into low- and high-risk subgroups, and ARGPs risk scores were calculated as follows: risk score = [value of ATG4B|CHMP4C×(-0.31)] + [value of CHMP2B|MAP1LC3B×(0.30)] + [value of CHMP6|RIPK2 ×(-0.33)] + [value of LRSAM1|TRIM5×(-0.26)] + [value of MAP1LC3A|PAFAH1B2×(-0.15)] + [value of MAP1LC3A|TRIM21×(-0.08)] + [value of MET|MFN2×(0.38)] + [value of MET|MTDH×(0.47)] + [value of RASIP1|TRIM5×(-0.23)] + [value of RB1CC1|TPCN1×(0.22)]. Nomograms were generated to better assess the ability of the ARG risk score to predict the 1-, 2-, and 3-year survival rates of patients (**Figure 2E**). By calculating the total risk score, oncologists could easily obtain the OS probability predicted by the nomogram for an individual patient. The correlations between ARGs in the model were constructed as a PPI network in the STRING database and mapped using Cytoscape software (**Figure 2F**).

### Prognostic Value of the ARG-Based Risk Model in the TCGA-PAAD Dataset

**Figure 3:**
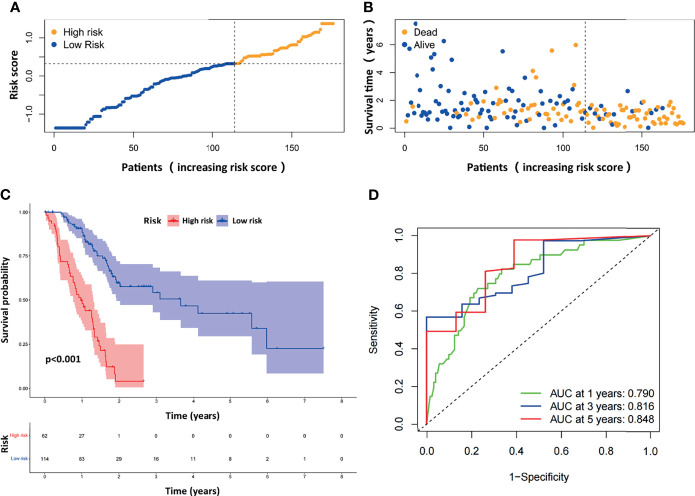
Evaluation of the prognostic ARGPs in the TCGA-PAAD dataset. **(A)** Distribution of risk scores of high- and low-risk PAAD patients based on the ARGPs-based prognostic signature. **(B)** The scatter plot showed the correlation between the survival status of high- and low-risk PAAD patients. **(C)** Kaplan-Meier analysis of the high-risk and low-risk groups of PAAD patients in the TCGA. **(D)** ROC curves of the risk scores of PAAD patients in the TCGA at 1, 3, and 5 years. AUC, area under the receiver operating characteristic curve.

The distribution of the risk scores and survival statuses was then analyzed by ranking the risk scores (**Figures 3A, B**). The results showed that patients with higher risk scores were more likely to be deceased. Examination of the survival curves of the low-risk and high-risk patient groups was performed by the Kaplan-Meier method (**Figure 3C**). The AUCs for 1-year, 3-year and 5-year OS were 0.790, 0.816 and 0.848, respectively, in the TCGA-PAAD dataset (**Figure 3D**).

### Verification of the ARG-Based Risk Model in the GEO Merged Cohort

**Figure 4:**
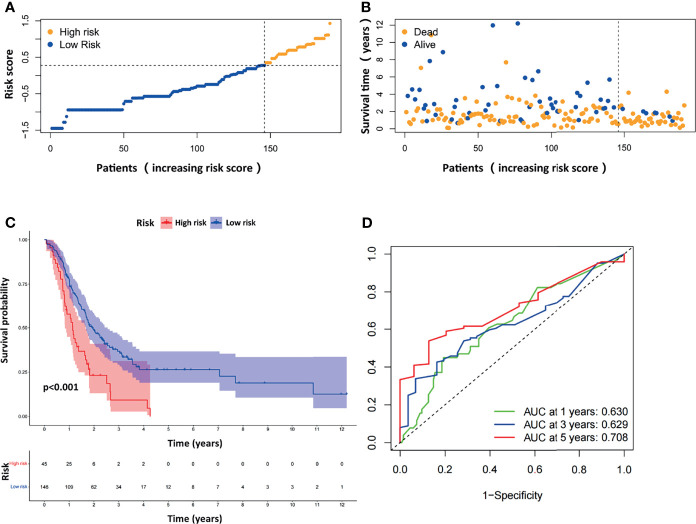
Evaluation of the prognostic ARGPs in the GEO-merged cohort. **(A)** Distribution of risk scores of high- and low-risk PAAD patients based on the ARGPs-based prognostic signature. **(B)** The scatter plot showed the correlation between the survival status of high- and low-risk PAAD patients. **(C)** Kaplan-Meier analysis of the high-risk and low-risk groups of PAAD patients in the GEO-merged cohort. **(D)** ROC curves of the risk scores of PAAD patients in the GEO merged cohort at 1, 3, and 5 years. AUC, area under the receiver operating characteristic curve.

We next evaluated the prognostic efficiency of the ARGP-based risk model by analyzing the data in pancreatic cancer cohorts from the GEO dataset. The distribution of the risk scores and survival statuses and the expression profiles of risk-associated ARGs were also analyzed by ranking the high-risk and low-risk pancreatic cancer patient groups in the GEO merged cohort according to risk scores (**Figures 4A, B**). Examination of the survival curves of the low-risk and high-risk patient groups was performed by the Kaplan-Meier method (**Figure 4C**). The AUCs for 1-year, 3-year and 5-year OS were 0.630, 0.629 and 0.708, respectively, in the TCGA-PAAD dataset (**Figure 4D**). Overall, the accuracy of the ARGP-based risk model was confirmed in the independent validation pancreatic cancer cohorts. Kaplan-Meier analysis and ROC curve analysis were also performed with GSE57495 and GSE78229. The results were similar to those of the GEO merged cohort and are shown in **Supplementary Figure 1**.

### Evaluation of the Diagnostic Efficiency of the ARGPs Risk Score

**Figure 5:**
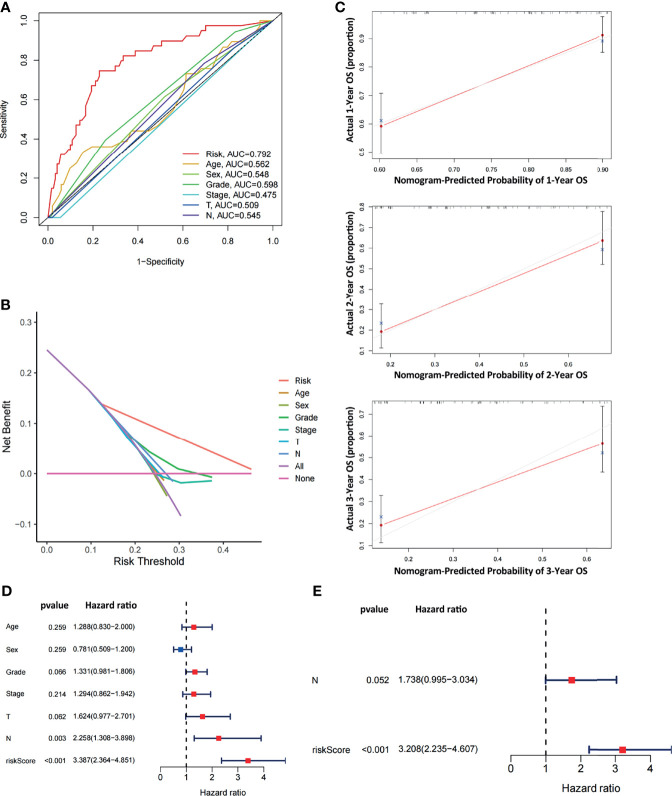
Comparative analysis of diagnostic efficiency between the risk score and clinicopathological characteristics. **(A)** AUC values of the risk score and clinicopathological characteristics. **(B)** DCA of the risk score and clinicopathological characteristics. **(C)** Calibration curves of the ARGP-based risk score in predicting the OS probability at 1/3/5 years in the TCGA dataset. **(D, E)** Univariate and multivariate analyses of prognostic factors in the TCGA cohort. T, N indicate T staging and N staging. The calculation of M staging is ignored due to too little data.

To comparatively evaluate diagnostic efficiency between the ARGP risk score and clinicopathological characteristics, ROC curves were generated for the risk score and clinicopathological characteristics, as shown in **Figure 5A** (The calculation of M staging is ignored due to too little data). The AUC of the ARGP risk score in PAAD patients was higher than those of the clinical indexes (AUC=0.792, 1 year). DCA was performed and further showed that the risk score served as a better prognostic indicator than other variables in clinical decision-making (**Figure 5B**). In addition, we used calibration curves to evaluate the prediction accuracy of the model (**Figure 5C**). The calibration curves showed that the predicted results of the ARGP model at 1, 2, and 3 years were close to the standard curve, which indicated that the model has good prediction performance.

Both univariate and multivariate analyses were performed to identify prognosis-related factors in TCGA pancreatic cancer patients (**Figures 5D, E**). Significant factors with p values < 0.05 in the univariate analysis were further included in the multivariate analysis. The results of the forest maps suggested that the risk score was the only significant factor after multivariate analysis. Therefore, the risk score calculated with 10 ARGPs was independently associated with the prognoses of patients (P<0.001, HR=3.208, 95% CI=2.235-4.607).

### Risk Gene Pairs and Clinical Characteristics Analysis

**Figure 6:**
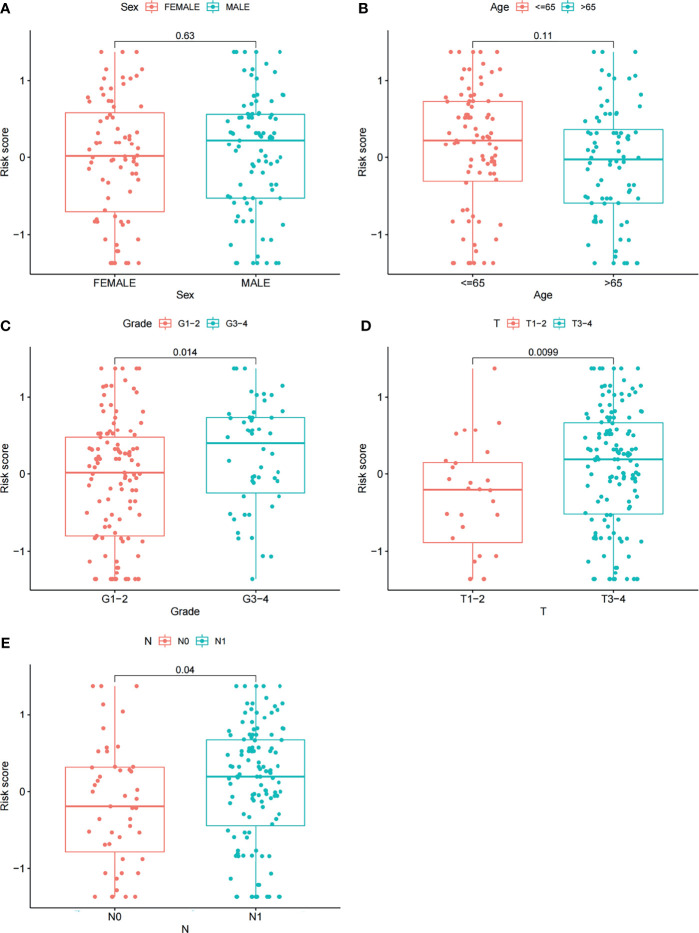
Correlations between risk scores and clinical features. The distribution of risk scores between the two groups was examined by sex **(A)**, age **(B)**, grade **(C)**, T stage **(D)** and N stage **(E)**.

Furthermore, we analyzed the relationships between clinical features such as sex, age, grade, T stage, N stage and the risk score. The relationship between the stage/M stage and risk score was not determined because the relevant information was unknown for most patients. The results are shown in **Figure 6** and revealed that there were large differences in risk scores between the grade, T-stage and N-stage groups (*P* < 0.05), indicating that our risk model was closely associated with the above clinical characteristics in PAAD.

### Differences in Infiltration of Immune Cells Between the High-Risk and Low-Risk Groups

**Figure 7:**
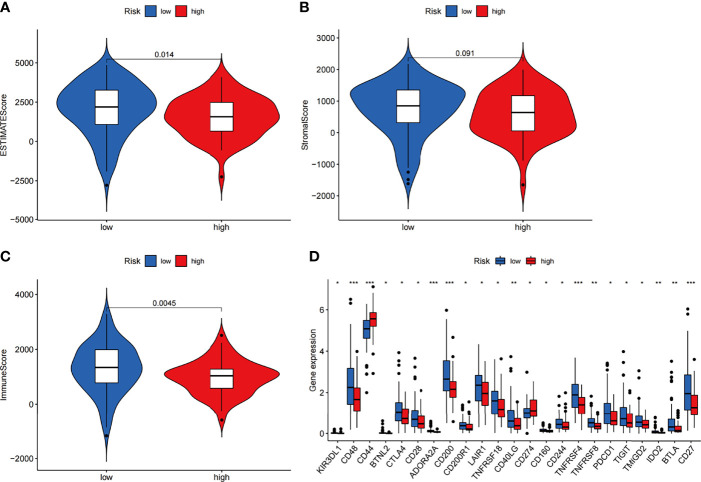
Estimation of immune cell infiltration in the two groups of the TCGA-PAAD cohort according to ARGP scores. **(A–C)** Violin plots of tumor purity for the low- and high-risk groups according to the stromal score **(A)**, immune score **(B)** and ESTIMATE score **(C)**. **(D)** Comparison of the expression of immune checkpoint genes between the high- and low-risk groups (*p < 0.05, **p < 0.01, and ***p < 0.001).

We next investigated how ARGs affected the prognoses of patients in a pancreatic cancer cohort. Considering that autophagy is reported to be associated with immune cell infiltration, which is known to correlate with cancer development and prognoses, we investigated the relationships between the proportions of immune and stromal components and the expression of ARGPs. The stromal score, immune score and ESTIMATE score were assessed in the low-risk and high-risk groups using the ESTIMATE R package. The results showed no difference in stromal scores (**Figure 7B**, p=0.091) but significant differences in immune scores (**Figure 7C**, p=0.0045) and ESTIMATE scores (**Figure 7A**, p=0.014) between groups. The expression of immune checkpoint genes is associated with the therapeutic effects of multiple immunotherapies. We thus investigated the differences in checkpoint genes between the two groups (**Figure 7D**). The results showed statistically significant differences between the two groups in the expression of all checkpoint genes, especially CD48, CD44, ADORA2A, CD200, TNFRSF4, and CD27 (P<0.001). Therefore, the results showed that the change in ARGP expression was associated with the TME and had an obvious influence on the expression of immune checkpoint genes.

**Figure 8:**
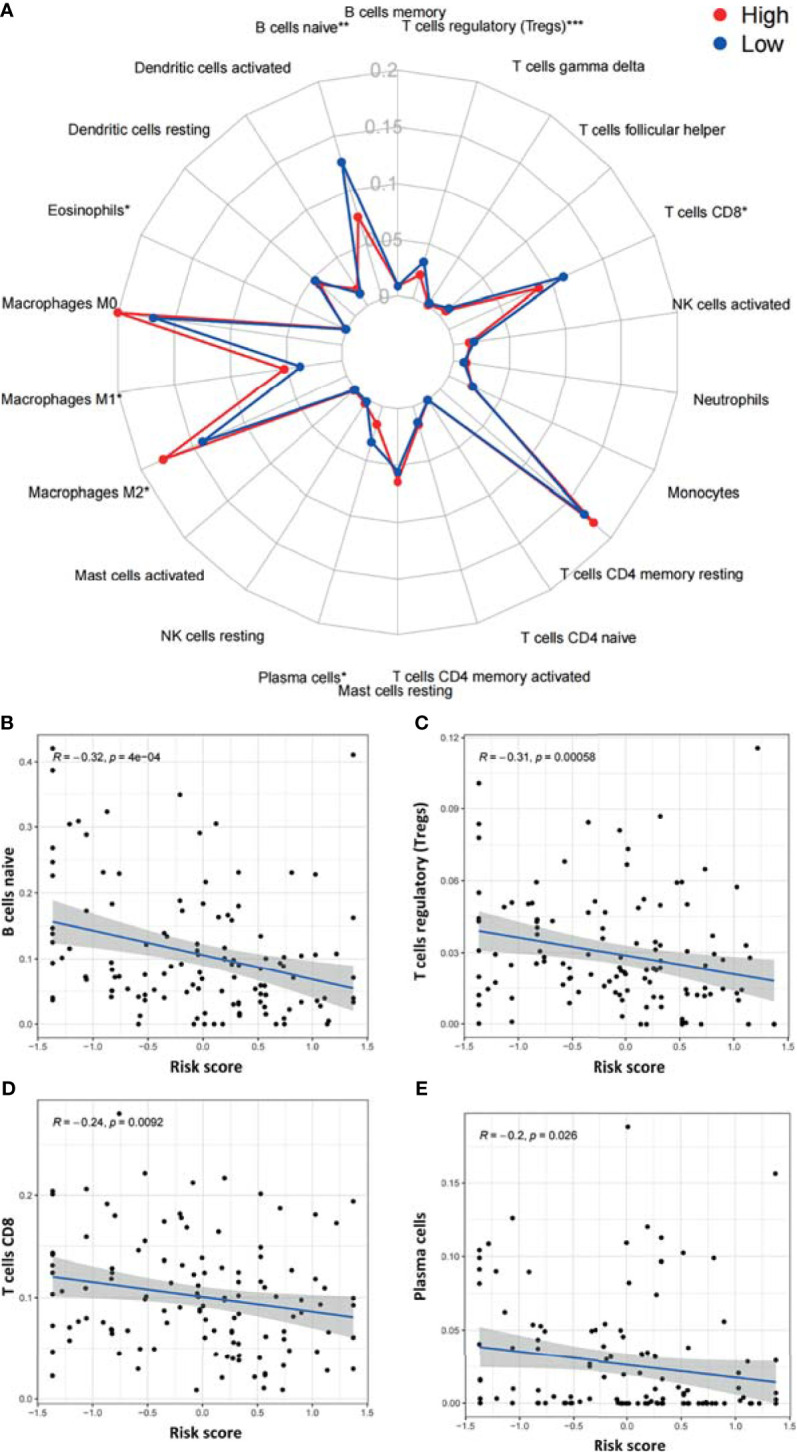
Correlations between the ARGP-based model and the infiltration of immune cells determined by CIBERSORT. **(A)** Differences in the infiltration of immune cells between the high‐ and low‐risk groups. **(B–E)** Correlations between the risk score and immune cells were further examined by Spearman correlation analysis, and results with significant differences are shown (*p < 0.05, **p < 0.01, and ***p < 0.001).

We next utilized CIBERSORT to examine differences in the abundance of 22 immune cell types between the high- and low-risk groups in TCGA-PAAD (**Figure 8A**). Among all 22 immune cell types, as many as six immune cell types were different between the two groups—the levels of naive B cells, Treg cells, CD8 T cells, and plasma cells were higher in the low-risk group, and the levels of M1 macrophages and M2 macrophages were higher in the high-risk group. In addition, the correlations between the risk score and six immune cells were further examined by Spearman correlation analysis, and the results showed that naive B cells (r=-0.32, p<0.001), Treg cells (r=-0.31, p<0.001), CD8 T cells (r=-0.24, p=0.0092), and plasma cells (r=-0.2, p<0.026) were statistically correlated with the ARGP risk score (**Figures 8B**–**E**).

### DEG-Based Functional Enrichment Analysis and Related Small-Molecule Drug Screening

**Figure 9:**
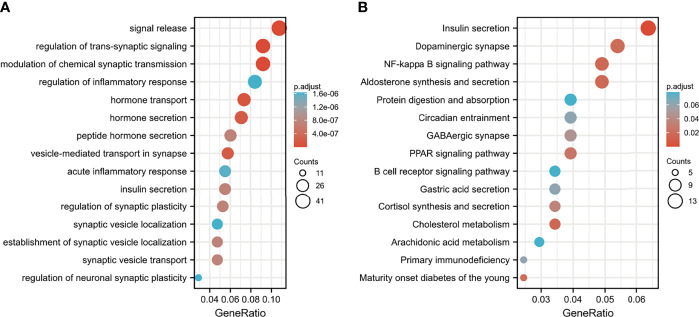
Functional enrichment analysis of DEGs between the low- and high-risk groups. **(A)** GO-BP analysis and **(B)** KEGG pathway analysis.

DEGs between the high-risk group and the low-risk group were further subjected to GO-BP analysis and KEGG analysis. The results showed that signal release, regulation of transsynaptic signaling, and modulation of chemical synaptic transmission were the top three enriched BPs (**Figure 9A**), while insulin secretion, dopaminergic synapses, and the NF-kappa B signaling pathway were the top three enriched KEGG pathways (**Figure 9B**).

**Figure 10:**
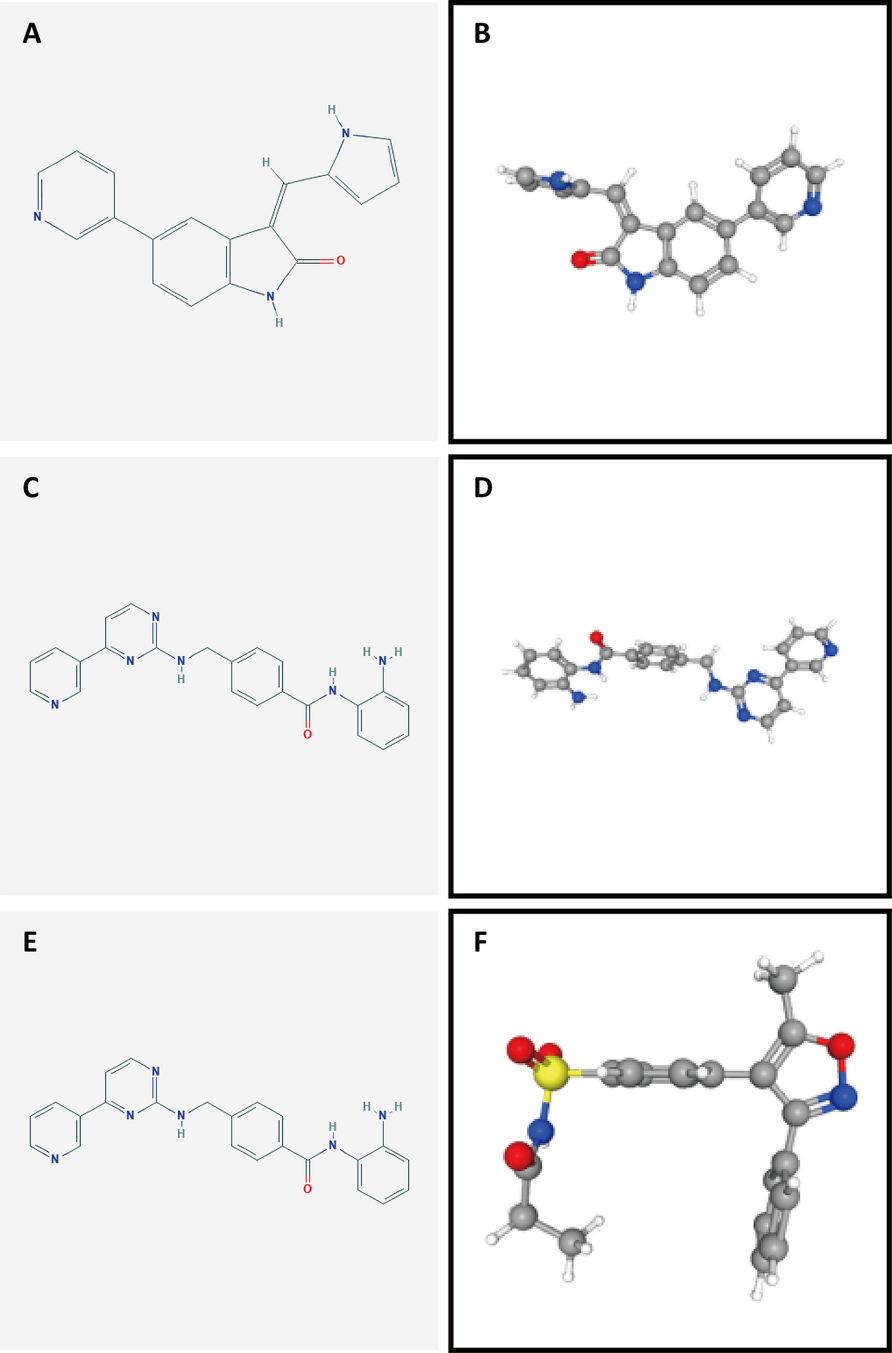
Structures of the screened small-molecule compounds. DEGs were uploaded to the L1000FWD website to screen for potential small-molecule compounds. The top three compounds were visualized using the PubChem website. **(A, B)** 2D and 3D structures of JAK3-inhibitor-VI. **(C, D)** 2D and 3D structures of mocetinostat. **(E, F)** 2D and 3D structures of parecoxib.

To identify potential drugs for PAAD, we uploaded the upregulated and downregulated DEGs to the L1000FWD website and matched them with small-molecule therapies. **Table 2** lists the 10 most significant small-molecule drugs and their similarity scores. The drugs with the top three negative fractions were JAK3-inhibitor-VI, mocetinostat and parecoxib, which were predicted to reversely regulate the expression of DEGs. Their 2D and 3D conformations were visualized using the PubChem website (**Figures 10A–F**). These potential small-molecule drugs may reverse the induction of gene expression by autophagy, providing guidance for the development of targeted drugs for PAAD. Further studies are still needed to confirm the value of these candidate small molecules in PAAD treatment.

**Table 2:** The screened drugs for PAAD treatment.

Drug	Similarity score	p-value	q-value	Z-score	Combined score	MOA	Predicted MOA
JAK3-inhibitor-VI	-0.0954	6.3E-12	3.85E-08	1.69	-18.9	Unknown	PARP inhibitor
mocetinostat	-0.0883	2.38E-10	0.000000392	1.73	-16.61	HDAC inhibitor	CDK inhibitor
parecoxib	-0.0848	8.63E-10	0.000000998	1.73	-15.68	cyclooxygenase inhibitor	cyclooxygenase inhibitor
BRD-K41220170	-0.0848	1.2E-09	0.00000128	1.76	-15.66	Unknown	protein synthesis inhibitor
ISOX	-0.0848	2.54E-09	0.00000209	1.77	-15.21	Unknown	HDAC inhibitor
desloratadine	-0.0813	7.54E-09	0.00000442	1.7	-13.84	histamine receptor antagonist	histamine receptor antagonist
kavain	-0.0813	9.15E-10	0.00000102	1.74	-15.74	Unknown	cyclooxygenase inhibitor
RS-504393	-0.0813	1.66E-09	0.00000158	1.85	-16.25	CC chemokine receptor antagonist	adrenergic receptor antagonist
lestaurtinib	-0.0777	2.39E-08	0.0000104	1.64	-12.51	FLT3 inhibitor, growth factor receptor inhibitor, JAK inhibitor	adrenergic receptor antagonist
BRD-K48576794	-0.0777	1.99E-09	0.00000177	1.76	-15.33	Unknown	cyclooxygenase inhibitor

## Discussion

Pancreatic cancer is a highly aggressive malignancy with a very poor prognosis and a 5-year survival rate of less than 10% (1). Despite the fact that methods for the detection and treatment of malignant tumors have rapidly advanced, early diagnosis and treatment strategies for PAAD are unsatisfactory. Recently, a study reported the role of ARGs in PAAD (24); however, these results lacked verification in external datasets. This is particularly important for PAAD because the sample size in each available database is small. In this research, a rank-based prognostic model was established based on ARGs from the TCGA PAAD cohort. The model was validated in multiple datasets (GSE57495, GSE78229, and GSE85916) in this study.

After we translated the commonly expressed ARGs into ARGPs, we performed a series of analyses based on the TCGA dataset. Finally, 10 ARGPs were identified to build a LASSO Cox prognostic model for PAAD patients, and a risk score was calculated for each patient. The final risk score was defined as [value of ATG4B|CHMP4C×(-0.31)] + [value of CHMP2B|MAP1LC3B×(0.30)] + [value of CHMP6|RIPK2 ×(-0.33)] + [value of LRSAM1|TRIM5×(-0.26)] + [value of MAP1LC3A|PAFAH1B2×(-0.15)] + [value of MAP1LC3A|TRIM21×(-0.08)] + [value of MET|MFN2×(0.38)] + [value of MET|MTDH×(0.47)] + [value of RASIP1|TRIM5×(-0.23)] + [value of RB1CC1|TPCN1×(0.22)]. Compared with traditional studies of gene signatures, rank-based methods are capable of overcoming the batch effect on various platforms and show no need for the scaling and normalization of data. Therefore, we merged three pancreatic cancer cohort datasets from the GEO database to obtain a larger PAAD cohort for validation (n=191). The results from the TCGA dataset and multiple external datasets support that the ARGP risk score plays an important role in the prognoses of PAAD patients. Furthermore, we compared the diagnostic efficiency of the risk score and clinicopathological characteristics. The results showed that the ARGPs risk score was significantly related to prognoses in the PAAD cohort and was proven to be an independent prognostic factor by univariate and multivariate Cox regression analysis. Correlation analysis showed that the risk scores of patients with late-stage cancer were higher, indicating that the scores were closely related to tumor progression. Overall, these findings suggested that our signature could potentially improve treatment in the PAAD cohort.

A total of 10 autophagy-related gene pairs were included in our model, of which CHMP2B|MAP1LC3B, MET|MFN2, MET|MTDH, and RB1CC1|TPCN1 were regarded as risk factors, while ATG4B|CHMP4C, CHMP6|RIPK2, LRSAM1|TRIM5, MAP1LC3A|PAFAH1B2, MAP1LC3A|TRIM21, and RASIP1|TRIM5 were protective factors. In the PPI network, MAP1LC3A/B (microtubule-associated protein 1 light chain 3 alpha/beta), which is also known as LC3A/B, was a central molecule among the included autophagy-related genes and is commonly used as a biomarker to detect autophagy (**Figure 2F**) (36). Mechanistically, LC3 is mainly involved in the formation of autophagosomes. Microtubule-associated protein LC3 is translocated from the cytosol to the isolation membrane of the nascent autophagosome (37, 38). In addition, MET|MFN2 and MET|MTDH were two ARGPs with the largest coefficients. MET is a tyrosine kinase receptor with one well-established ligand, hepatocyte growth factor (HGF) (39). The HGF/MET axis is involved in a series of biological responses, such as proliferation, angiogenesis, migration, invasion, metastasis, and survival, thus contributing to tumorigenesis, development, and progression in different human cancer types (40, 41). In addition, the HGF-MET axis regulates mTOR activity and controls serum starvation-mediated autophagy and biogenesis (42) In pancreatic ductal adenocarcinoma, several studies have reported that MET promotes malignant phenotypes and contributes to tumor growth (43, 44). Therefore, MET serves as a promising target in the treatment of cancer (41, 45–47), as well as a prognostic marker of clinical value. MFN2 (mitofusin 2) is a novel controller of mitophagy activation, which is an essential housekeeping process required to maintain tumor homeostasis (48, 49). Several studies have reported antitumor effects of MFN2 in different malignancies, including gastric cancers, breast cancer, hepatocellular carcinoma and urinary bladder cancer (50–52). In pancreatic cancer, MFN2 has been demonstrated to induce autophagy through inhibition of the PI3K/Akt/mTOR pathway. Therefore, overexpression of MFN2 may provide an effective treatment strategy in pancreatic cancer (53). MTDH (metadherin) is known to induce multidrug resistance gene 1 (MDR1) expression and participates actively in autophagy and chemoresistance (54). Recent research reported that MTDH-stimulated 5-FU chemoresistance may be mediated through autophagy activated by the AMPK/ATG5 pathway (55).

Unlike most solid tumors, the activity of programmed cell death 1 receptor (PD-1)/programmed cell death ligand 1 (PD-L1)- and cytotoxic T-lymphocyte-associated protein 4 (CTLA4)-blocking agents against PAAD is disappointing. Few novel combinations of immunotherapeutic agents reported to date have achieved satisfactory results (56, 57). Considering that autophagy plays a major role in the differentiation and homoeostasis of immune cells and is required for the development and maturation of most immune cell types of both myeloid and lymphoid lineages (58, 59), we next investigated the relationship between ARGPs and immune activity in PAAD patients. ESTIMATE analysis showed a lower level of immune cell infiltration in the TME of the high-risk group of PAAD patients. Notably, the investigation of expression of checkpoint genes also showed obvious differences between the two groups, which indicates that the ARG-based model may provide potential targets for PAAD treatment.

Furthermore, we utilized CIBERSORT to analyze differences in immune cell subpopulations between the high- and low-risk groups. The results showed that naive B cell, Treg cell, CD8 T cell, and plasma cell levels were lower in the high-risk group and further showed statistically significant correlations with the risk score. Complex aggregates of cytotoxic lymphocytes, B lymphocytes (including plasma cells) and dendritic cells are considered tertiary lymphoid structures (TLSs) (60). The presence of TLSs in tumor tissue has largely been associated with favorable prognoses in patients with solid tumors (61). Several studies have demonstrated that cytotoxic CD8^+^ T cells are important effector cells in adaptive immunity that specifically recognize and clear tumor cells and are thereby associated with improved survival in cancer patients (62). Previous studies found that the infiltration of CD20^+^ B cells in ovarian cancer, non-small-lung carcinoma and cervical cancer was correlated with improved survival and lower relapse rates (62, 63). TLSs were proven to contain large aggregates of plasma cells positively associated with the antitumor response. Similar to our model, two recent prognostic models in PAAD revealed that low levels of B cells (plasma cells) and CD8^+^ T cells were associated with poor prognoses in pancreatic cancer (64, 65). Interestingly, we also noticed that several immune infiltration analyses of ARGs in other cancers obtained similar results, especially higher levels of naive cells in low-risk groups (66–68). The relationship between B cells and autophagy in cancers is worthy of further research. Regulatory T cells are highly dependent on autophagy and are one of the populations most vulnerable to autophagy inhibition/depletion (18). It has been demonstrated that autophagy enforces the functional integrity of regulatory T cells by coupling environmental cues and metabolic homeostasis (69). In conclusion, ARGPs can reflect the degree of infiltration of immune cells and provide clues for studying the mechanism by which autophagy affects immune function in pancreatic cancer.

We then performed GO-BP and KEGG enrichment analyses of DEGs. KEGG analysis comparing the low-risk and high-risk groups showed that DEGs were mainly enriched in insulin secretion pathways. It has been reported that autophagy is involved in insulin resistance following endoplasmic reticulum stress in diabetes (70) and differentially regulates insulin production and insulin sensitivity (71). Interestingly, retrospective studies have shown a survival improvement in diabetic patients with many solid tumors, including pancreatic cancer, who have been treated with metformin (common drug for diabetes mellitus type 2 that is reported to enforce autophagy) compared with patients treated with insulin or sulfonylureas (72–74). GO-BP analysis showed that signal release, the regulation of transsynaptic signaling, and the modulation of chemical synaptic transmission were also enriched in DEGs, and these processes may be involved in the regulation of autophagy *in vivo*.

We further investigated potential small-molecule drugs with significant down expressed genes. Even though many of these drugs are not used clinically, comparison of the functions of drugs targeting differentially expressed ARGPs may reveal putative therapeutic biomarkers for further validation because gene expression alterations in cancer can influence treatment outcomes (75). Among the top three drugs, mocetinostat has been identified as an HDAC inhibitor. Malignant amplification of tumor cells is characterized by unchecked cellular proliferation and high genetic instability. Upregulated HDAC (histone deacetylase) enzyme activity is associated with closed chromatin assembly and subsequent inhibition of gene expression, indicating a characteristic feature of malignantly transformed cells (76). As an HDAC inhibitor, mocetinostat has been reported to enforce tumor antigen presentation, reduce immune-suppressive cell types and benefit checkpoint inhibitor therapy (77). In addition, parecoxib has been identified as a cyclooxygenase inhibitor that effectively enhances radiation sensitivity in colorectal cancer cells (78). However, the exact functions of the listed small-molecule drugs warrant further investigation.

There are also several limitations in our research. Firstly, the vast majority of patients in TCGA cohort are in stage 2 (detailed in **Supplementary Table 3**). The reason may be that the early-stage patients have not found the disease. While the terminal cancer patients have not chosen surgery, which made it difficult to obtain clinical samples in both early and later stage. Therefore, the construction process of the model may have selection bias that is difficult to correct, leading to a potential limitation that this algorithm might not predict outcomes across all pancreatic cancer. Besides, further biology experiments are needed to improve the understanding of autophagy-related mechanisms in pancreatic cancer.

In conclusion, the identified risk-associated ARGPs could provide a basis for the development of PAAD therapeutic interventions with autophagy-related mechanisms. Of importance, the predictive value of the signature was verified in multiple external databases, indicating that this novel risk model can robustly classify PAAD patients into low- or high-risk groups, further providing a reference for the formulation of precise treatment plans. Nevertheless, further prospective experiments are required to confirm the clinical value of this model in defining optimal personalized targeted treatments and to explore treatments targeting ARGs in PAAD.

## Data Availability Statement

The datasets presented in this study can be found in online repositories. The names of the repository/repositories and accession number(s) can be found in the article/**Supplementary Material**.

## Author Contributions

QZ collected the papers, analyzed the data, analyzed the conclusions, and drafted the manuscript. LL reviewed the data and conclusions. PM and Yangyang Z contributed to manuscript writing. JD and Yanyu Z presented the idea for this manuscript, supported the funding, analyzed the conclusions, and drafted and revised the manuscript. All authors contributed to the article and approved the submitted version.

## Funding

This study was funded by the National Natural Science Foundation of China (Grant No. 81902055).

## Conflict of Interest

The authors declare that the research was conducted in the absence of any commercial or financial relationships that could be construed as a potential conflict of interest.

## Publisher’s Note

All claims expressed in this article are solely those of the authors and do not necessarily represent those of their affiliated organizations, or those of the publisher, the editors and the reviewers. Any product that may be evaluated in this article, or claim that may be made by its manufacturer, is not guaranteed or endorsed by the publisher.
