# Supplementary material for: Identification of an Autophagy-Related Pair Signature for Predicting Prognoses and Immune Activity in Pancreatic Adenocarcinoma
Source: Front Immunol. 2021 Dec 9;12:743938. doi: 10.3389/fimmu.2021.743938 (PMC8695429; doi:10.3389/fimmu.2021.743938)
Supplement: Supplementary file 1 [file DataSheet_1.docx]

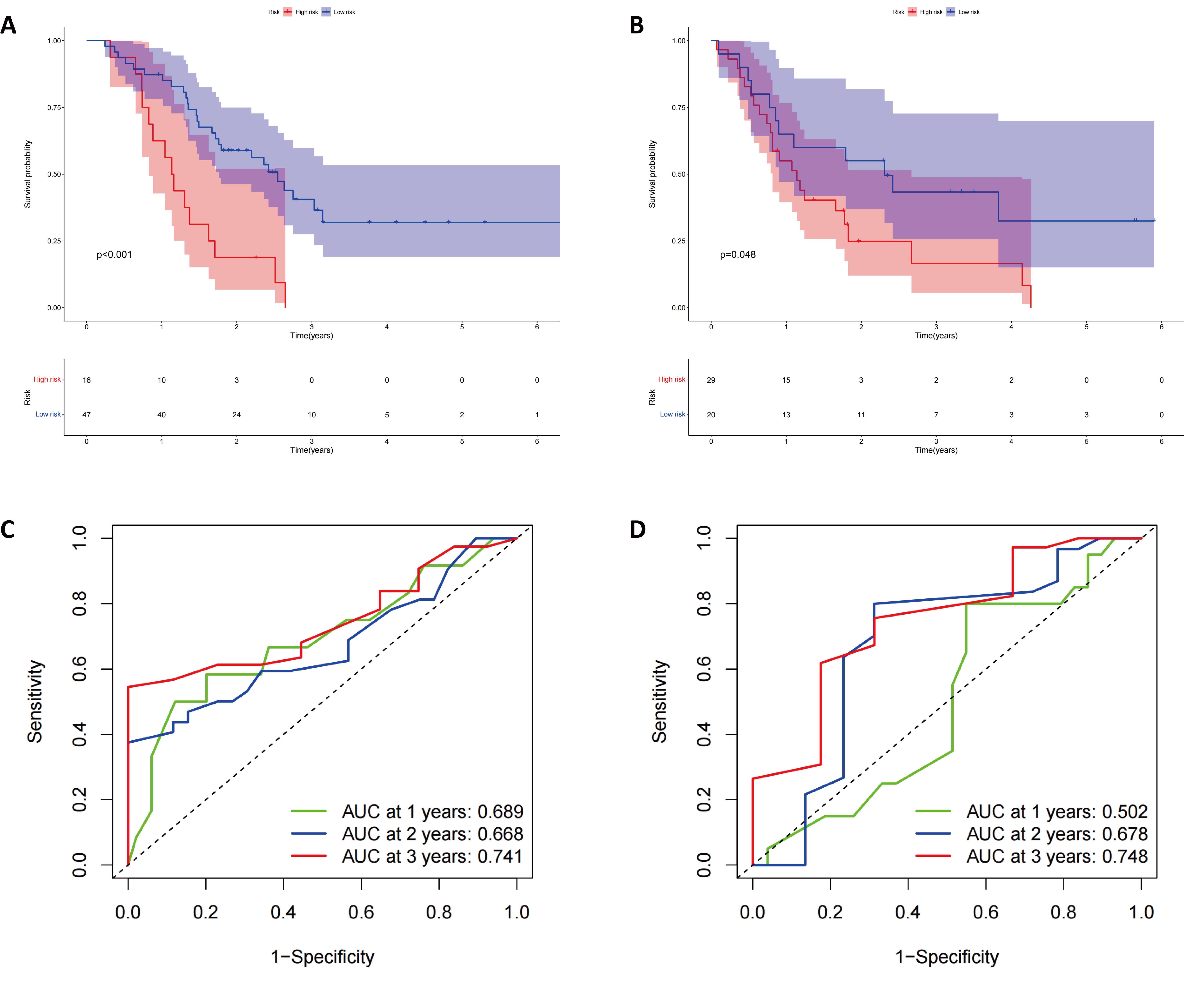


**Supplementary Figure Survival Analysis of the PAAD Cohort.** Kaplan-Meier analysis of the high-risk and low-risk groups of PAAD patients in the GSE57495 (A), and GSE78229 cohorts (B). The ROC curves of the risk score of PAAD patients in the GSE57495 (C), and GSE78229 cohorts (D). AUC: The area under the receiver operating characteristic curve.
